# Spontaneous Subtrochanteric Femoral Stress Fracture Related to Alendronate : A Case Report

**DOI:** 10.5704/MOJ.1303.002

**Published:** 2013-03

**Authors:** Paul CC Chew, B Julaihi, ZA Ibrahim

**Affiliations:** Orthopaedic Clinic, Normah Medical Specialist Centre, Kuching, Malaysia; Radiology Clinic, Normah Medical Specialist Centre, Kuching, Malaysia; Department of Pathology, Universiti Malaysia Sarawak, Kuching, Malaysia

## Abstract

Spontaneous atypical fractures of the femur have been reported in patients on long-term antiresorptive bisphosphonate therapy. Here, we report a case of subtrochanteric stress fracture in a seventy-year-old female patient on long-term alendronate therapy, and accompanying management challenges. Potential measures to prevent this complication of antiresorptive treatment for osteoporosis include the following: setting strict criteria for prescribing antiresorptive therapy, limiting the duration of continuous antiresorptive therapy, and increasing the use of bone anabolic agents.

**Key Words:**

atypical femoral fractures, alendronate, osteoporosis

## Introduction

Recently, there has been an increase in the number of reports describing atypical femoral diaphyseal and sub-trochanteric fractures related to long-term usage of antiresorptive drugs for the treatment of post-menopausal osteoporosis^1^,^2^. Alendronate, a bisphosphonate, the most commonly used antiresorptive drug was implicated in a few reports. Here, we report a case of stress or insufficiency fracture in the subtrochanteric region of the left femur in a female patient who was prescribed alendronate for the previous six years, highlighting the uncommon presentation and radiological appearance of such fractures.

## Case Report

A seventy-year-old female presented with two months' history of left anterior thigh pain. She was not able to walk for more than fifteen minutes because of the pain, but reported no pain at rest and no history of trauma. Her medical history included hypertension and hypercholesterolemia for which atenolol and lovastatin were prescribed. Physical examination showed scoliosis of the lumbar spine, 'flat back', a positive Trendelenburg test and ambulation with a marked limp. The patient had no complaints of back pain on movement of the spine and the straight-leg-raising test was normal bilaterally. Hip and knee movements were pain free. Differential clinical diagnosis included referred pain from spinal pathology and pain arising from the left hip or femur or thigh.

**Fig. 1:**
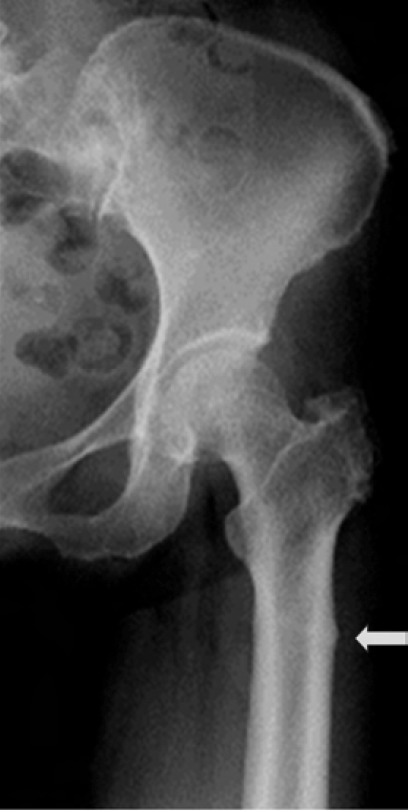
: Radiograph of left hip showing a subtle periosteal reaction (arrow) in the subtrochanteric region of the left femur.

**Fig. 2a & 2b:**
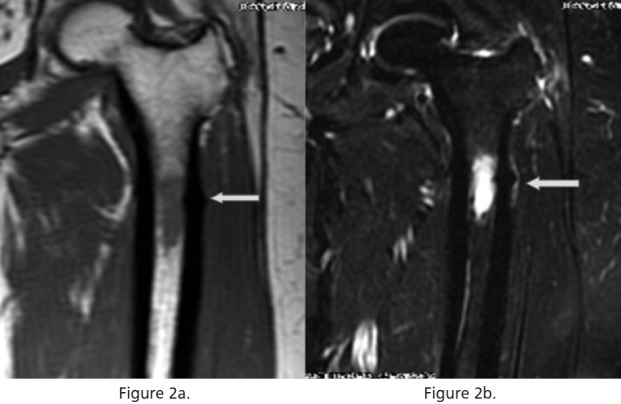
: T1 weighted and T2 weighted magnetic resonance images of upper femur showing signal changes in the marrow at the level of the periosteal reaction.

**Fig. 3a & 3b:**
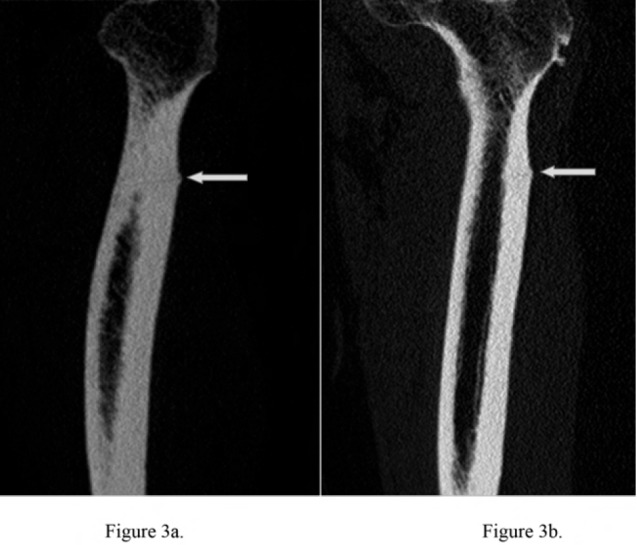
: Figure 3a and 3b are reconstructed images from a limited multislice CT scan of the left femur showing a thin oblique linear fracture in the upper femur. There was no marrow lesion seen.

Radiographs of the lumbosacral spine showed degenerative scoliosis with spondylotic changes at multiple levels. Radiographs of the pelvis showed very subtle thickening of the lateral cortex in the sub-trochanteric region of the left femur (Figure 1) and normal hip joints. Magnetic resonance imaging (MRI) of the lumbosacral spine showed disc degeneration at multiple levels with spinal canal stenosis at L4/5 and L5/S1. MRI of the left femur revealed a hypointense area in the subtrochanteric marrow in the T1 weighted image and hyperintensity on the T2 weighted image. (Figure 2a, 2b); there was no further enhancement with gadolinium. Differential diagnosis for such MRI findings included stress fractures or osteomyelitis. Limited computed tomography (CT) scan of this area confirmed a linear fracture at the lateral cortex of the proximal femur. (Figure 3a, 3b).

On further questioning, the patient revealed that she had been taking alendronate for six years. Her general practitioner prescribed alendronate treatment for chronic backache. Dual energy X-ray absorptiometry (DEXA) scan was not performed prior to or during treatment. A diagnosis of alendronate-related stress fracture of the proximal left femur was made.

DEXA scan (Hologic Discovery) results were normal, with a T-score of 0.6 for lumbar spine L1 to L4 and T-score of 1.0 for the right hip. Serum calcium, 25-hydroxy Vitamin D level and intact parathyroid hormone levels were also normal. Serum Beta-CrossLaps, a bone resorption marker and an indicator of effectiveness of antiresorption therapy of bisphosphonates, was normal at111ng/L. We do not have pre-alendronate treatment results.

Prophylactic intramedullary nailing was performed after ascertaining that the patient’s femoral medullary canal could accommodate a nail. The medullary canal was reamed to thirteen millimeters to accommodate a ten millimeter nail.

Insertion of the nail at the lower end of the femur was difficult due to anterior bowing of the femur; this resulted in conversion to a complete sub-trochanteric fracture. We then implemented dynamic hip screw fixation to stabilize the fracture (Figure 4a). Tightening of the screws further extended the fracture. Curetted bone material from the defect in the greater trochanteric region was sent for histopathologic analysis (Figure 5).

Bisphosphonate therapy decreases the number or function of osteoclasts by promoting apoptosis or inhibits osteoclast action in bone remodeling, thereby inducing a progressive increase in bone density. Stress fractures are caused by micro-crack accumulation in bone that has not undergone sufficient remodeling^3^. As expected, due to the bisphosphonate therapy, we saw no osteoclasts in specimen sent for analysis, indicating a lack of ongoing bone remodeling. The sample (Figure 5) showed areas of necrotic bone with empty lacunae (black arrow) adjacent to viable bone (blue arrow) and foci of micro-cracks seen at the necrotic bone fragments (green arrow).

Alendronate treatment was stopped and the patient was started on recombinant parathyroid hormone (rPTH or Forteo, Eli Lilly) therapy. Follow-up x-rays showed union of the fracture (Figure 4b), and by that time the patient could walk without pain.

## Discussion

Bisphosphonates have been shown to reduce rates of spinal and extra-spinal fragility fractures in patients with prior osteoporotic fractures. However, concerns exist about the effects of long-term suppression of bone resorption and remodeling on bone strength. Although this is reflected in many reports of atypical femur fractures in patients on longterm bisphosphonate therapy, large population studies have not shown conclusive evidence of direct causation^4^,^5^. The number of reported fractures is too small and the variance too wide to allow authors to draw definitive conclusions.

The following reported figures are cause for concern. In a review of 12,777 women aged 55 years or older with fractures of the femur in Sweden in 2008, 59 patients with atypical femoral fractures were identified. Of the 59 fractures, 46 were long-term bisphosphonate users (crude incidence of 5.5 fractures per 10,000 patient-year) 5. Of additional concern, bisphosphonates are now more widely used due to the availability of cheaper generic equivalents, and increasingly affluent society and increased public awareness of the importance of osteoporosis treatment.

The present case involved an uncommon presentation of a stress fracture with minimal changes in plain radiography. CT Scan was more useful to detect breaks in cortical continuity, while MRI clearly showed intra-medullary and periosteal bone changes. Further, this case illustrates the fact that osteoporosis treatment must be instituted appropriately and monitored for possible complications. Alternative strategies such as intermittent bisphosphonate therapy or drug ‘holidays’ should be considered. Alternating antiresorptive treatment with a bone anabolic agent like rPTH is also an attractive alternative, but the high cost of rPTH and the need for daily subcutaneous injections limit widespread adoption of this strategy. In stress fractures, decisions regarding the mode of internal fixation can be challenging.

**Fig. 4a:**
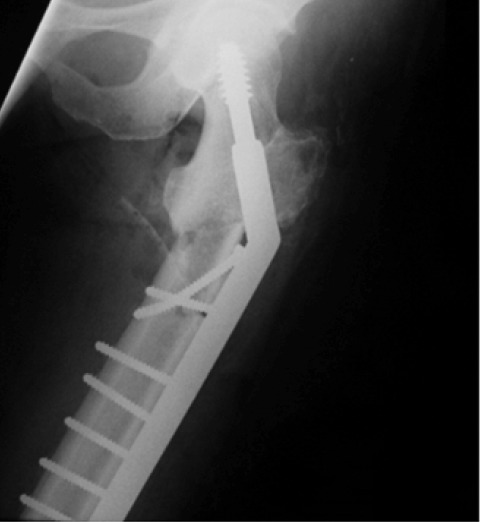
: Radiograph showing dynamic hip screw fixation of the subtrochanteric fracture

**Fig. 4b:**
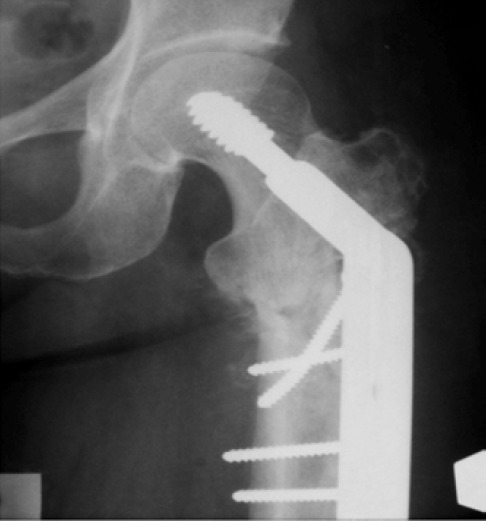
: Radiograph taken two months postoperatively showing healing callus formation

**Fig. 5:**
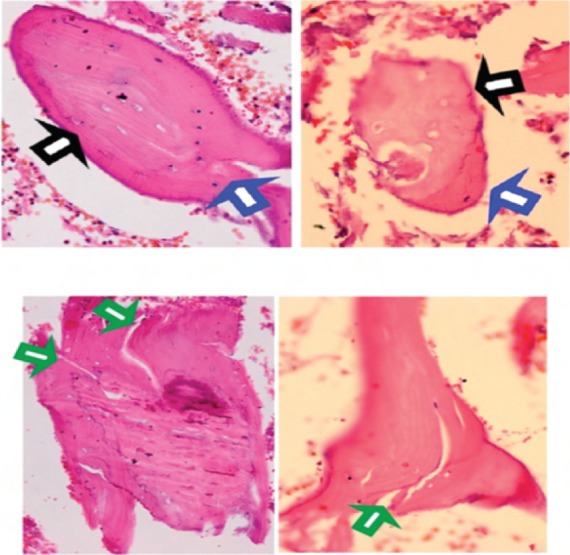
: Histology of bone from the left greater trochanter. (Haematoxylin and Eosin (H&E) staining, 40X magnification). Areas of necrotic bone with empty lacunae indicated by black
